# Temporal variability in shell mound formation at Albatross Bay, northern Australia

**DOI:** 10.1371/journal.pone.0183863

**Published:** 2017-08-30

**Authors:** Simon J. Holdaway, Patricia C. Fanning, Fiona Petchey, Kasey Allely, Justin I. Shiner, Geoffrey Bailey

**Affiliations:** 1 Anthropology, School of Social Sciences, the University of Auckland, Auckland, New Zealand; 2 Department of Environmental Sciences, Macquarie University, New South Wales, Australia; 3 Department of Archaeology, University of York, King's Manor, York, United Kingdom; 4 Waikato Radiocarbon Laboratory, Hamilton, New Zealand; 5 Archaeology, Flinders University, South Australia, Australia; New York State Museum, UNITED STATES

## Abstract

We report the results of 212 radiocarbon determinations from the archaeological excavation of 70 shell mound deposits in the Wathayn region of Albatross Bay, Australia. This is an intensive study of a closely co-located group of mounds within a geographically restricted area in a wider region where many more shell mounds have been reported. Valves from the bivalve *Tegillarca granosa* (Linnaeus, 1758) were dated. The dates obtained are used to calculate rates of accumulation for the shell mound deposits. These demonstrate highly variable rates of accumulation both within and between mounds. We assess these results in relation to likely mechanisms of shell deposition and show that rates of deposition are affected by time-dependent processes both during the accumulation of shell deposits and during their subsequent deformation. This complicates the interpretation of the rates at which shell mound deposits appear to have accumulated. At Wathayn, there is little temporal or spatial consistency in the rates at which mounds accumulated. Comparisons between the Wathayn results and those obtained from shell deposits elsewhere, both in the wider Albatross Bay region and worldwide, suggest the need for caution when deriving behavioural inferences from shell mound deposition rates, and the need for more comprehensive sampling of individual mounds and groups of mounds.

## Introduction

**Fig 1:**
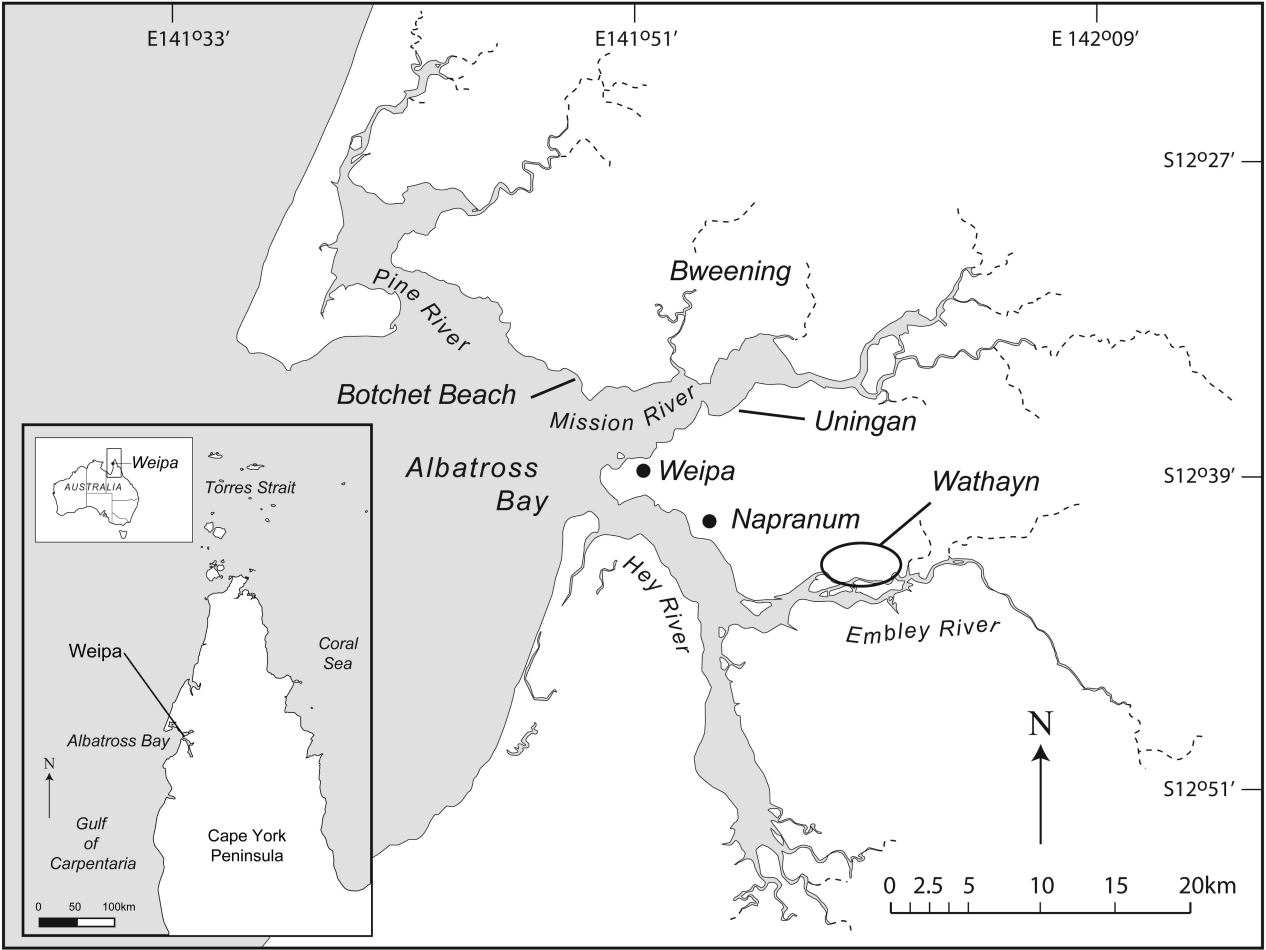
Location map of the study area. Albatross Bay is a shallow, semi-circular embayment on the northwestern coast of Cape York Peninsula in far north Queensland, Australia, that opens into the Gulf of Carpentaria. The study area of Wathayn is situated on the northern side of the Embley River, one of four estuaries draining into Albatross Bay. The map also shows the location of other places mentioned in the text. (Modified from [30] under a CC BY license, with permission from Elsevier, original copyright 2016).

**Fig 2:**
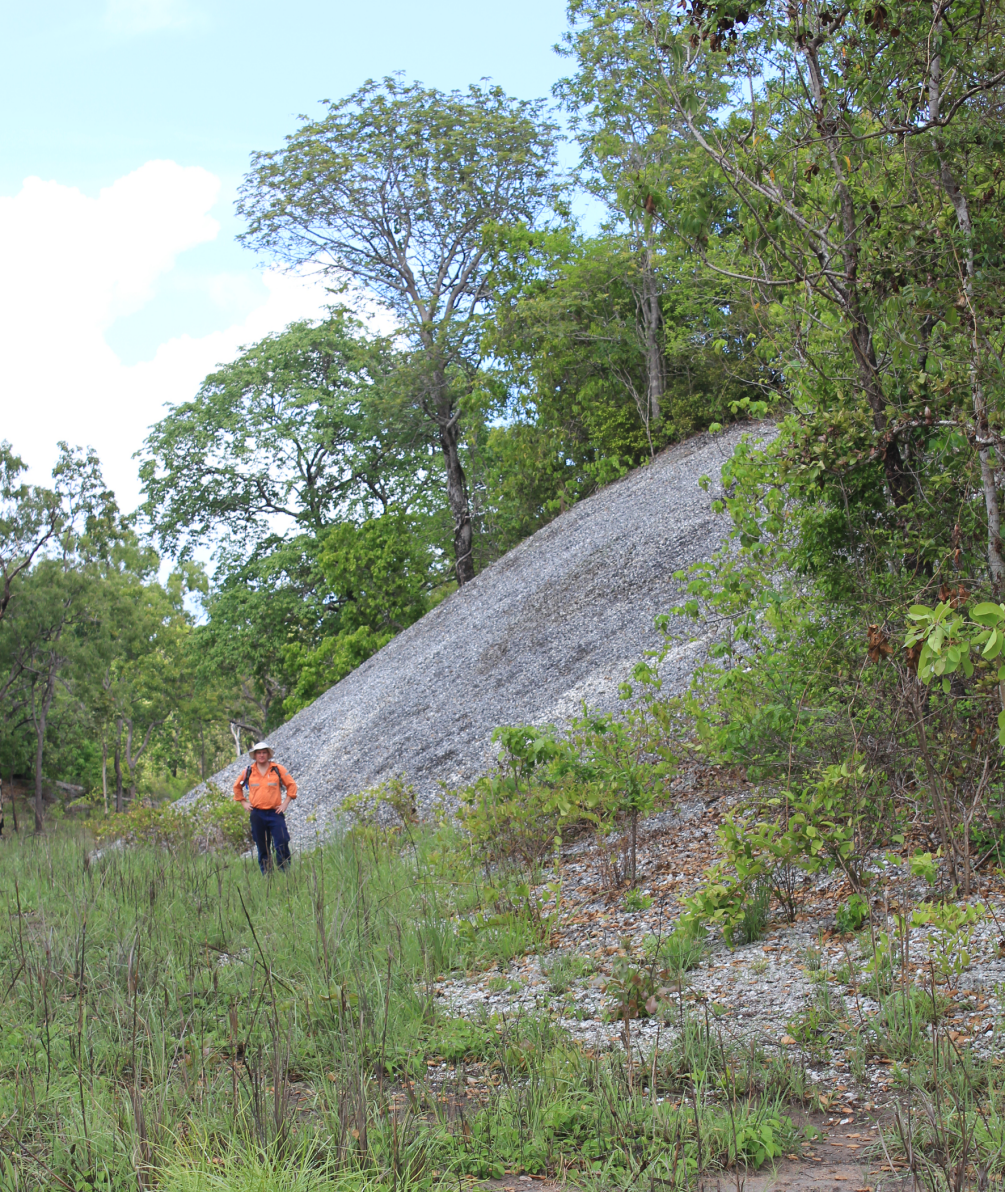
Large shell mound in the vicinity of the study area. Predominantly composed of the shells of the bivalve *Tegillarca granosa* (syn. *Anadara granosa* (Linnaeus 1758)), some mounds attain heights of ten metres or more. (Source of image: PCF. The individual pictured gave written informed consent (as outlined in PLOS consent form) to publish his image).

The region of Albatross Bay on the western coast of Cape York Peninsula, northern Australia (Fig 1), contains a record of both coastal and inland human occupation. Several hundred shell matrix deposits (SMD), characterized by a predominance of shells of the bivalve *Tegillarca granosa* (syn. *Anadara granosa* (Linnaeus 1758)), have been recorded near to the tidal estuaries of the four rivers (Pine, Mission, Embley, and Hey Rivers) that feed into the bay (Fig 1). Large mounded SMDs are a feature of the archaeology of many coastal and aquatic habitats around the world. We define mounded SMDs as midden deposits, i.e. deposits of food debris created by human activity, in which the shells form the majority of the sedimentary matrix of the deposit resulting in visible features in the landscape [1]. Both as a consequence of their composition and because of their prominence in the landscape (Fig 2), these features are often singled out for attention–the “mound phenomenon” [2]. In Australia, for instance, they are commonly referred to as shell mounds [2, 3, 4] while in South Africa they are termed megamiddens [5]. In California, Lightfoot and Luby [6] list the terms shell mounds, shell middens, shell scatters, and shell heaps. In Brazil they are known as *sambaquis* [7, 8, 9].

Here we use results of an intensive study of a sample of shell mounds from one area within Albatross Bay to consider some of the issues involved in dating these types of deposits. In many situations, archaeologists are limited by the amount of material suitable for dating. For example, charcoal preservation often places severe constraints on where samples for radiocarbon age determination may be obtained [10]. The same is not true of shell mounds where, because the shell itself can be dated, there is potentially an abundance of samples. To be sure, there are issues, discussed further below, that need to be addressed concerning the conventional radiocarbon age (CRA) and the dates assigned to mound formation episodes but, in principle, since the shell itself can be dated, samples are always present. This raises questions about how samples should be selected, and what we might expect the radiocarbon results to show.

We tackle these questions in the context of reporting the results of 212 radiocarbon determinations from the excavation of 70 SMDs in the Wathayn region of Albatross Bay (Fig 1). These are particularly large sample sizes, in terms of both the number of SMDs excavated and sampled and also the total number of radiocarbon determinations obtained in a single geographic location, compared with other studies on *T*. *granosa* SMDs in the Albatross Bay region, or across northern Australia. For example, Faulkner [11, 12] obtained 39 age determinations from 20 SMDs in the Blue Mud Bay area of northeast Arnhem Land, on the western side of the Gulf of Carpentaria, while Rosendahl et al. [13] reported 17 age determinations from 14 shell mounds on Mornington Island in the southwest of the same region. On the Abydos coastal plain of northwestern Australia, Clune and Harrison [14] reported that *T*. *granosa* shell mounds were initiated sometime between 4,400 and 5,300 BP, and while mounds appear to have ceased forming some 1,800–1,600 years ago, middens continued to form until the early twentieth century or later. These conclusions were based on eight determinations from two shell mounds and a smaller deposit referred to as a midden. Similarly, small sample sizes have characterized previous work on the Weipa deposits. Here we analyse the impact of greatly increased numbers of radiocarbon determinations on interpretation of the chronology of mound formation.

## Previous work at Weipa

Bailey [2, 15, 16, 17] described over 500 SMDs around Albatross Bay and its tributaries in the northern Cape York region, the location for this study. The largest of these were over 10 m high and estimated to contain up to 10,000 tonnes of shell, although the majority were smaller mounds less than 1 m thick. The mounds were associated with a mangrove-lined estuarine environment: some were located on tidal mudflats within or behind the mangrove barrier, while others were located on low sandy chenier ridges on the inner margin of tidal mudflats or on the beach front; still others were situated well back from the tidal margin, on the bauxite plateau that slopes gently up from the river [2]. The dominant shell species was the bivalve, *T*. *granosa*, accounting for ~95% by weight of all shell material. Other mangrove and mudflat species were present but in very small quantities, along with small quantities of fish and marsupial bones, bone and stone artefacts recovered from one excavation sample at the Kwamter mound, and deposits of charcoal. Radiocarbon dates bracketed the formation of the mounds to the past two millennia and the youngest date is 235±110 radiocarbon years BP (I 1737) from the Kwamter mound, consistent with use up until the period of European contact and corresponding with Roth’s [18] observation of camp fires on top of one of the Weipa mounds. The base of the Kwamter mound, at the mouth of the Embley River, was dated to 1,180±80 radiocarbon years BP (SUA 149). Stone subsequently obtained radiocarbon dates from Kwamter covering a similar age range, and Beaton obtained radiocarbon age determinations from other Weipa shell mounds, extending the age range back another 1,000 years [19, 20, 21].

In summarising the then record of shell mound formation around the Gulf of Carpentaria and Cape York Peninsula, Bailey [2] noted that *T*. *granosa*-dominated shell mound formation was confined to the last one to two millennia, and that there was a considerable hiatus (up to 4,000 years) between the stabilisation of sea level following the last post-glacial rise and the initiation of shell mounding activity. That *T*. *granosa* were present in the region at least 4,000 years ago was, however, attested by shell midden deposits in the Walaemini rockshelter at Princess Charlotte Bay on the eastern side of Cape York Peninsula [2].

Since then a number of additional studies have been conducted on the Weipa shell mounds, by Morrison [22, 23, 24, 25, 26] and by the authors [27, 28, 29, 30]. Morrison [25] summarised and analysed all known, published radiocarbon determinations on anthropogenic SMDs and small shell scatters in the Albatross Bay catchment, a total of 93 determinations from 48 sites along the margins of the Pine, Mission, Embley, and Hey Rivers (Appendix 1 and 2 and Fig 2 in [25]). All conventional radiocarbon ages were calibrated with CALIB v.6.10 using data from IntCal143/SHCal13 and Marine13 calibration curves. The dataset indicated that SMD formation spans almost the entire 200–2,700 cal BP period. However, there are very few sites with ages older than 1,500 cal BP, with the result that a summed probability distribution of the determinations peaks at 500–700 cal BP (Fig 9 in [25]). Morrison used these data to argue that broader continental models positing a cessation of mound-building across northern Australia after 500–700 cal BP might not apply to Albatross Bay, but acknowledged that selective sampling might be an issue and that “…there is a clear need for more detailed occupation chronologies for multiple shell matrix deposits within specific study areas to develop more robust, regional chronologies…” (pp. 11 in [25]).

The research reported here is one such study. The sampling technique we adopted, described below, allows us to discuss the timing of shell mound formation at a single geographic location, and to consider the variability in rates of accumulation over time. The relatively broad spatial sample of mounds we studied also allows us to comment on variability in the places where mound formation occurred. But while our sampling strategy provided a great deal of this information, it also imposed limits on what we could infer from the dates we obtained. The implications of these limits will also be discussed.

## Expectations for SMD formation

The marine bivalve mollusc *T*. *granosa* inhabits intertidal areas over much of the Indo-Pacific region (pp. 390 in [31]). While the molluscs have been found inhabiting areas of sandy mud, the highest densities are found on substrates of soft intertidal to marginally subtidal muds bordering mangrove forests near, but not in, the mouths of large rivers [32]. Studies show that they appear to tolerate relatively wide fluctuations in water salinity, from lows of 5–10 ppt to highs of 31 ppt (pp. 6 in [32]), though feeding rates drop off as salinity declines. *T*. *granosa* do not burrow into the mud to any depth, and are often found lying with the posterior end protruding above the surface. They are therefore relatively easy to access by foragers. However, while they are hyper-abundant, they produce little edible meat per shell. For example, Pathansali and Soong (pp. 30 in [33]) report the proportion of boiled meat to whole shell weight for *T*. *granosa* of between 14.83 and 17.33%, with a meat weight per individual of between 1.86 and 4.05 g. As long as large numbers of molluscs can be obtained at one time, energy returns in relation to energy expended in gathering are likely to be adequate [34].

Nevertheless, bulk harvesting poses a number of logistical issues. First, because of the high shell-weight to meat-weight ratio and the need to heat the shells to remove the meat and make it palatable, containers such as bags are essential, and the energetic cost of transportation is high, imposing a strong incentive to find a location for processing the shells as close as possible to the harvesting area. Canoes or boats may improve the efficiency of transportation and increase the distance to the processing location.

Processing poses a second issue, for even if collecting the molluscs is relatively simple, opening large numbers to gain access to the meat is best done centrally, with the aid of fire. Heat opens the bivalves, so placing them in or near a fire greatly aids the efficiency of meat extraction. This process is succinctly described by Bailey (pp. 137–138 in [16]) and is also described by Hardy et al. [35] for present-day SMD formation in Senegal.

Finally, once the meat is extracted, there is the issue of shell disposal. Because of the relatively small size of the bivalves and the low meat-weight to shell-weight ratio, a given weight of meat generates a large quantity of shell. The simplest and most energetically efficient way to dispose of the shells is to discard them where the molluscs are processed, and there are reports by Aboriginal people today that the mounds were places where the shells were opened and the extracted meat then taken elsewhere (pp. 259 in [36]). In the Senegal example mentioned above, the extracted shell meat is dried at the processing location where the shells are discarded, and then traded over considerable distances.

The literature on SMDs contains a range of explanations as to why large-volume deposits–shell mounds–were formed. Some authors attribute the concentration of shells to the remains of intensive food extraction supporting, perhaps, large populations of people [37, 38]. Sampson [39] summarises arguments put forward by a number of authors that large concentrations of shell might result from feasting. Bailey (pp. 138 in [16]), on the other hand, estimates that the vast quantities of shell in the Weipa mounds (196,000 tonnes or about nine thousand million molluscs) could easily have been accumulated by repeated Aboriginal occupation over a period of as little as 100 years without invoking either significant population increase, including ceremonial gatherings, or technological change. SMDs are regarded by some as specialist processing sites [40] or simply as highly visible sites that represent only one component of a varied economy [2, 16]. Attention has also focussed on the size of the mounds and, at times, their shapes, with suggestions that they represent deliberate constructions–a form of monument [41, 42]. In some parts of the world, mounds are associated with large numbers of burials [7, 43]. In Australia, ethnohistoric accounts from Nukukadambal and Castlereagh Bay in Arnhem Land [44] indicate that people sometimes used earth and shell mounds as living platforms. In western Cape York, Wik Monkan people at times used elevated areas such as raised beaches for habitation during the wet season (late December-March) (pp. 215 in [44]).

These varied functions are not mutually exclusive, but how shell mounds were formed and for what purposes does have an impact on the chronology of mound formation, and hence on subsequent interpretations of mound function. Whatever the purpose of shell mounds, the rate of shell deposition, at least in the case of *T*. *granosa*, was likely always to have been high and variable, a function of the large volume of material that must be processed to gain a sufficient quantity of food. This has several important implications for sampling, the more so when it is the voluminous material (i.e., the shell) that provides the datable sample.

Firstly, if shells are deposited in large volumes, the rate at which shell deposition occurs will appear to be rapid. This is because each depositional event, or set of events, will create a large volume of deposit. Therefore, there is some chance that samples selected from different depths within a deposit will, in fact, date the same event (or a temporally equivalent set of events). This is, of course, also possible in non-shell matrix deposits, but is less likely to occur for samples separated in depth unless the volume of material deposited within a short period of time is also high. This means that the chronology of an SMD may look quite different to the chronology of another type of deposit when the same sampling protocol (based on, say, so many samples per depth of deposit) is employed. Hausmann and Meredith-Williams [45] have recently published a novel method, using oxygen isotope analysis coupled with intensive radiocarbon dating, for investigating this issue in rapidly accumulating mounds.

Secondly, it may be difficult to detect gaps in the depositional history of SMDs and therefore to infer either continuity of occupation or periods of abandonment. Inferences of continuous occupation are often made on the basis of uniform rates of deposition. If, for instance, sediment continues to be deposited in a cave or rockshelter at a reasonably constant rate and this sediment contains cultural material, then it is reasonable to suggest that occupation was continuous relative to the rate at which sediment was deposited. In principle, the same is true of shell mounds. However, because of the relatively large volume of shell deposited in one or a closely time-related set of discard events, the rate at which a shell mound accumulates may appear to be very rapid, and thus may appear to reflect continuous occupation. Cessation of deposition or abandonment may appear to be less common, dependent to a very large degree on the number of samples that are dated in a vertical sequence. In reality, of course, the episodic nature of shell deposition as a measure of the occupational history of the people who deposited the shells is dependent on the temporality of the processes by which material accumulates.

Thirdly, shell mounds that look similar may in fact have quite different depositional histories. Because the volume of shell discarded at any one time is relatively large, small differences in the number of depositional events on different mounds may lead to marked differences in the volume of material deposited. Put another way, a small number of depositional events, each involving a large number of shells, may potentially create a deposit that has similar dimensions to one that represents the accumulations from many depositional events where the amount of material deposited each time was smaller. Where large volumes of deposit are created, one could easily imagine situations where gaps in the depositional record are missed by the particular sampling procedure employed, especially where mounds appear to be stratigraphically homogeneous [46]. Occupation in such situations would appear continuous even when it was not. Of course, in principle, any of these situations are discoverable but only if every SMD is investigated, with a great deal of attention given to the differential rates at which volumes of shell accumulated. It is important to consider how different histories of deposition potentially leading to similar sized features, or indeed similar histories leading to different sized features, makes selection of SMDs for investigation highly problematic, at least based only on the criterion of size [28].

Fourthly, when accumulation rate is measured as the amount of material deposited over a particular period of time it is assumed that time appears only in the denominator of the calculation. However, shell may be subject to diagenesis dependent on local environmental variables [30] (following common usage in archaeology we use the term diagenesis to refer to both physical and chemical changes in shells as described in Chapter 3 of Claassen [1]). These processes lead to changes in the thickness of a shell deposit as the structure of the shell mound changes. If thickness is time-dependent because of post-depositional morphological changes in the deposit, interpreting rates of deposition becomes more complex. It may not, for example, reflect the rate at which shell was originally deposited but rather a combination of deposition and deformation. We discuss the issues that this raises in the analyses below.

At the beginning of our research in Albatross Bay, we recalibrated and assessed all the radiocarbon dates available at the time [27]. The oldest SMD dates then available came from Idholga on the Hey River, followed by those from Lueng on northern side of the Mission River (Fig 1), where the dates clustered into two periods (ca. 1,800–1,000 and 600–100 cal BP). SMDs in the lower reaches of the Mission and Embley Rivers were no older than 1,000 cal BP and the available evidence suggested that the most recent mound building occurred around 150 years ago (pp. 58–60 in [26]). These observations suggested that the development of the muddy substrates and shallow intertidal environments suitable for *T*. *granosa* beds, described above, had occurred in the upper regions of the estuarine rivers by at least 1,800 years ago, and that SMD formation might relate to a coastal evolution model that would have the estuaries infilling with sediment from upstream to downstream following attainment of the maximum Holocene sea level 7–6,000 years ago [26]. As discussed below, this hypothesis is not sustained by the results obtained from the shell mounds we studied in our Wathayn (Embley River) study area. However, this led us to consider in greater detail the way mounds might have accumulated, and the types of behavioural inferences in general that could be drawn from studying shell matrix deposits.

## Methods

Permission for this research to be conducted on Aboriginal land around Wathayn was provided by the Western Cape Communities Coexistence Agreement (WCCCA) and the Napranum Aboriginal Shire Council on behalf of the Traditional Owners.

Our shell sampling strategy at Wathayn aimed to address two key questions:

1. What is the chronology of SMD formation within a single geographical area?
2. Is there any spatial pattern in shell matrix deposit formation within that area?

To do this, we needed to obtain multi-date chronologies from as many sequences as possible within time and budget constraints, and to ensure that the dated sequences came from shell matrix deposits over the full locational range. The locations of all of the SMDs at Wathayn were obtained by a variety of methods:

1. The database of SMD locations previously compiled by RioTintoAlcan (Weipa) Pty Ltd as part of their cultural heritage surveys;
2. Inspection of high resolution aerial images;
3. Aerial survey from a light plane; and
4. Intensive on-foot survey of the study area.

**Fig 3:**
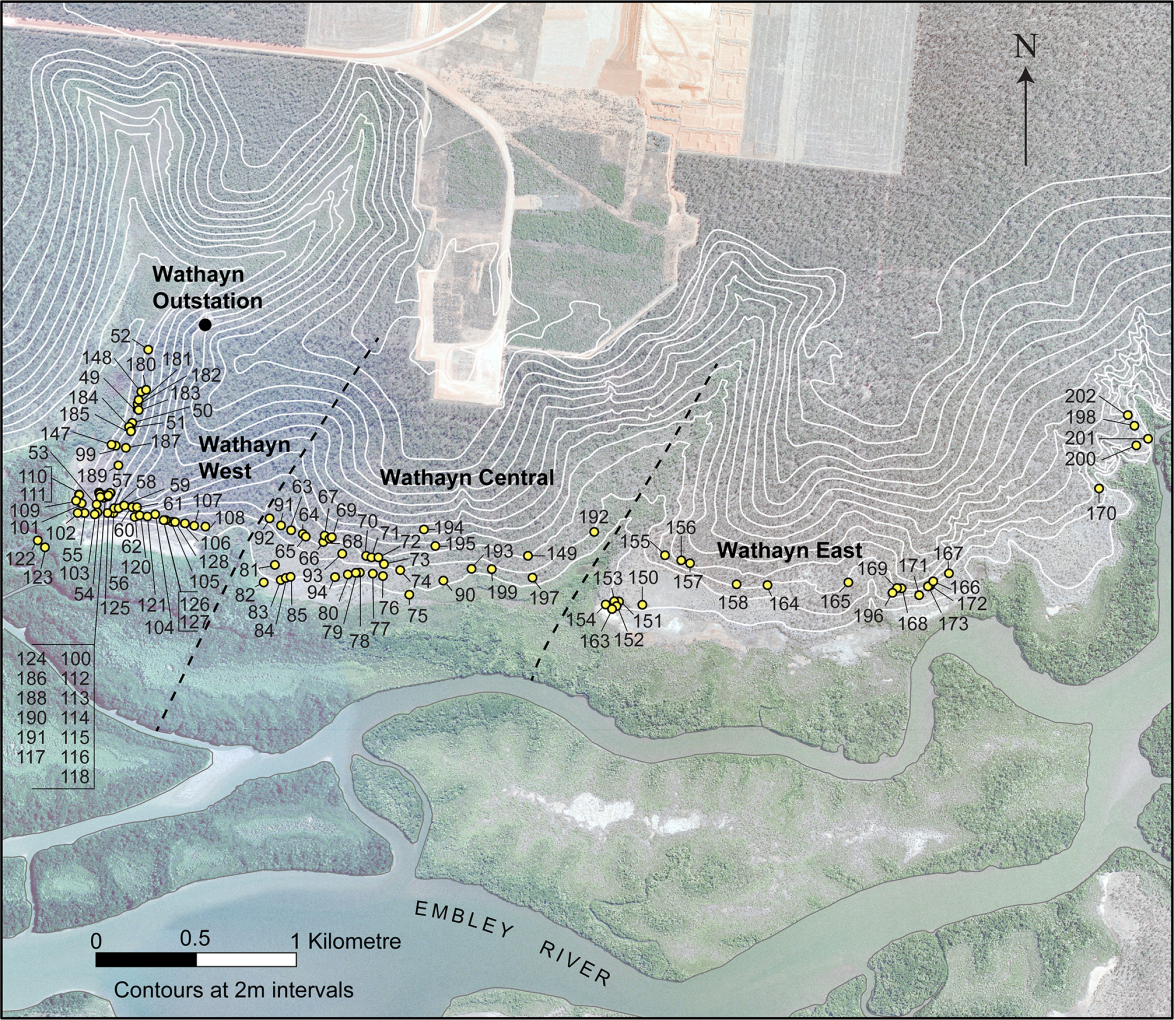
The Wathayn study area on the north bank of the Embley River, showing the locations of the shell mounds. Contours derived from airborne LiDAR data indicate the topography. Both LiDAR and air photography data provided courtesy of RioTintoAlcan (Weipa) Pty Ltd. (Modified from [30] under a CC BY license, with permission from Elsevier, original copyright 2016).

A total of 158 SMDs were located by a combination of these methods (Fig 3). The SMDs are located over a geographical range of 4.7 km east to west, or upstream to downstream, along the northern side of the Embley River, and within 1,200 m of the present day shoreline (Fig 3). They occupy a variety of geomorphic environments, ranging from higher elevation hillslopes and cliff tops to sand/gravel ridges and muddy estuarine floodplains low down in the landscape. For ease of subsequent analysis and discussion, the SMDs in the Wathayn study area have been grouped into three geographical locations (Wathayn East, Wathayn Central, and Wathayn West), separated from each other by tributary drainage depressions with no SMDs present (Fig 3).

**Fig 4:**
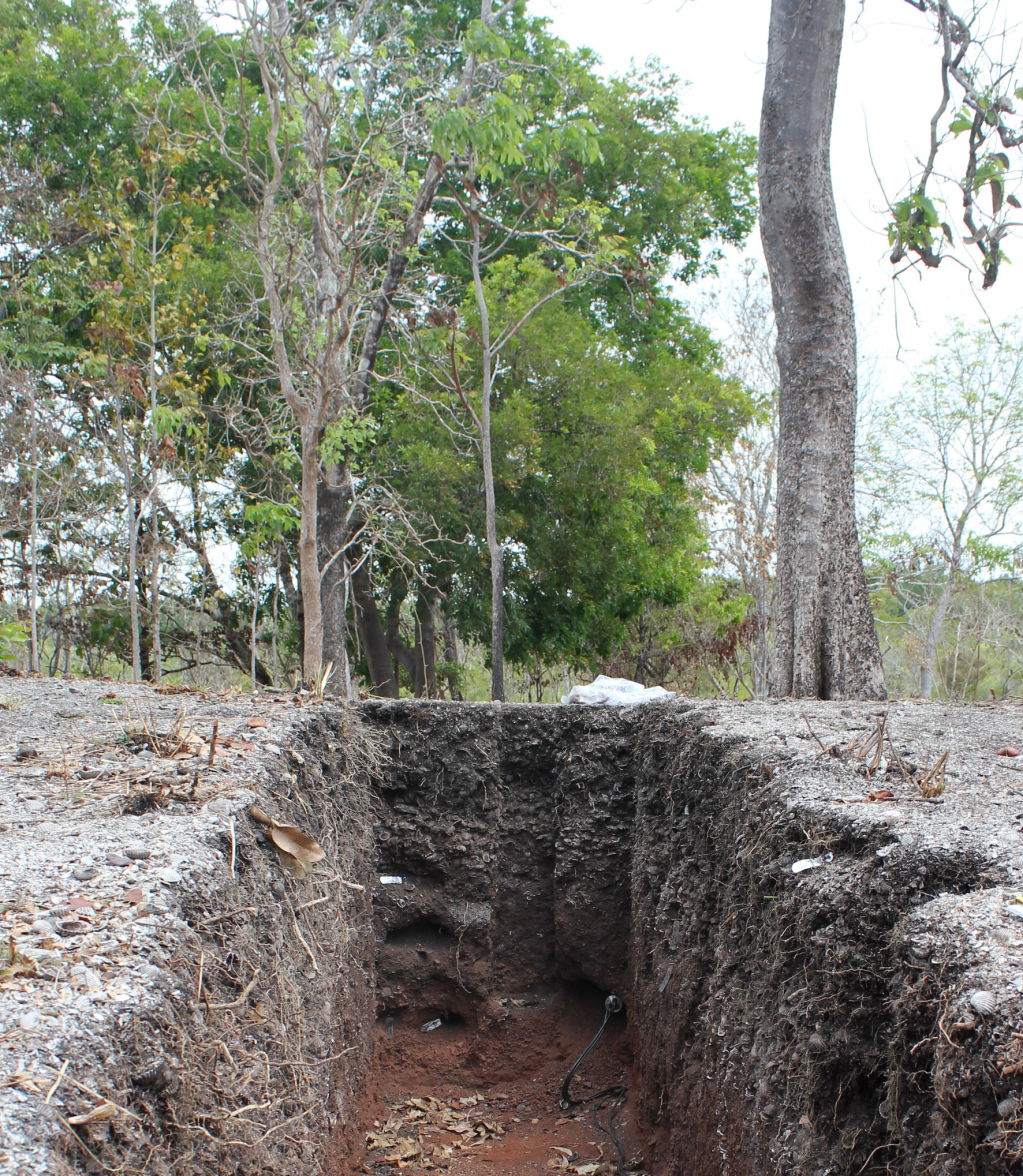
Trench dug by hand into WPSM55. The end face of the trench is located at the thickest part of the shell mound. Samples for radiocarbon dating, shell measurements, and Optically Stimulated Luminescence dating of the mound substrate have been collected.

Once located, all of the SMDs were trimmed of vegetation and surveyed using a Leica C10 Terrestrial Laser Scanner (TLS), with target locations fixed in 3D space using a Differential Geographic Positioning System (DGPS) [29]. To expose the internal stratigraphy and facilitate sample collection from the thickest part of the sequence, trenches 1 m wide were excavated by hand, using shovels, mattocks, and buckets, along the short axis to the centre of each SMD (Fig 4). The excavated material was stockpiled and used to backfill the trench and restore the mound surface at the end of each fieldwork season. This method was approved by the Indigenous Traditional Owners prior to excavation of the first SMD.

Shell and charcoal samples were selected from observable stratigraphic units within each of 49 SMDs, including the basal and uppermost deposits (n = 149). Additional samples were collected from the side walls of six of the trenches to try to document the within-mound chronological variability (n = 58). All of the samples were prepared and analysed at the Waikato Radiocarbon Laboratory in Hamilton, New Zealand, following standard radiometric and AMS radiocarbon protocols [47, 48]. The shells were washed in dilute HCl to remove surface contamination, and charcoal samples were treated with a series of dilute HCl and NaOH washes. All shells were tested for re-crystallization (alteration of CaCO_3_ from aragonite to calcite) prior to dating [49], and shells showing signs of alteration were rejected.

To obtain reliable calibrated results on *T*. *granosa* shells from the mounds, an initial test using three “paired” charcoal/shell determinations from the same stratigraphic units was undertaken to ascertain the local marine reservoir correction value (ΔR) for this region. All three ΔR results (Table 1) indicate enrichment in ^14^C compared to the average global ocean, as is often typical for estuarine shell species [28]. However, for archaeological ΔR, it is essential that the charcoal sampled comes from short-lived plants in contexts that are contemporaneous with the shell. Our pairs do not conform to these strict guidelines since all samples were highly weathered and could not be identified to short-lived species (Dr. Rod Wallace, wood identification expert at the University of Auckland, pers. comm. Nov. 2011). Additional age (i.e., inbuilt age which is the growth age of the tree) in charcoal will reduce the difference between shell and charcoal ^14^C determinations and the ΔR value will be smaller and less accurate. The values obtained for the Wathayn samples are slightly more negative than results from four historic pre-AD 1950 shells from the northwest Cape York Peninsula, reported by Ulm [50], which returned a pooled ΔR value of –103±16 ^14^C yrs. This latter correction figure was adopted for all of the calibrated age calculations reported here. Calendar ages were obtained using the Marine09 [51] and OxCal software v 4.1.7 [52].

**Table 1:** Paired charcoal/shell radiocarbon results to establish the marine reservoir correction value for this location.

Provenance (Mound WPSM number and depth)		Sample Material	^14^C age & error (BP) (Rs(t))	Marine modelled age (BP) (Rg(t))	ΔR (yrs) = Rs(t)–Rg (t)	Lab no.
75	135cm	Charcoal	1625 ± 25	1980 ± 19	–156±42	Wk 28977
	125cm	*Tegillarca granosa*	1824 ± 37	-		Wk 28976
76	160cm	Charcoal	2445 ± 25	2791 ± 81	–98±90	Wk 28985
	145cm	*Tegillarca granosa*	2693 ± 39	-		Wk 28984
77	190cm	Charcoal	2550 ± 25	2948 ± 27	–176±46	Wk 28992
	185cm	*Tegillarca granosa*	2772 ± 37	-		Wk 28991
ΔR Average					–158±29	

Delta R has been calculated following the method of Stuiver and colleagues [53].

Such a large database, of multiple calibrated radiocarbon age determinations in vertical stratigraphic sequence per mound, allows us to investigate in great detail the variability in shell accumulation rates both within and between mounds. Following the discussion above, however, we approached the analysis of deposition rate with caution.

We initially adopted the method of Stein and colleagues [54], who used the following formula to calculate rate of accumulation:

RA=TA/DA (1)

where RA = Rate of Accumulation in cm/year; TA = Total Accumulation, the difference in depth below the surface of dated sample pairs, in cm; and DA = Duration of Accumulation, the difference in radiocarbon years between the mean radiocarbon ages of the sample pairs. Like Stein and colleagues, we expressed the accumulation rate as depth per 100 years (calculations in S1 File). Other studies discuss methods for calculating rates of accumulation, including confidence intervals, with the goal of providing estimates for ages at different depths within a deposit (e.g. [55, 56, 57, 58]). These studies assume that the nature of deposition can be accurately modelled. However, as previously discussed, for SMDs deposition rate may be highly variable. Therefore, in this study, we calculated rates of deposition in a large number of deposits with the goal of documenting variability in deposition rate both through time and across space. The technique used, calculating rate of accumulation in centimetres per hundred years, uses only the mean calibrated radiocarbon ages between pairs of determinations. No consideration is given to possible changes in deposition rates within deposits between pairs of dates. If it cannot be assumed that deposition was relatively uniform between date pairs, calculating confidence intervals for deposition rates is problematic. The alternative is to consider the deposition rates in relative terms, as shown below.

## Results

### Mound ages

As indicated in Table 2, the shell mounds at Wathayn vary considerably in age, with the oldest determinations around 3,500–4,000 cal BP (for example, WPSM55) and the most recent ages within the last 500 years. A large number of mounds have calibrated ages around 2,000–2,500 cal BP (for example, WPSM80). Vertical sequences of radiocarbon determinations from some of the mounds are very close in age, with overlapping errors (Table 2). Others exhibit longer time spans of accumulation, a small number of which include what appears to be either a decreased level of shell discard or a hiatus in deposition (WPSM55, 63, 75, 81, 106, 151, and 152).

**Table 2:** Conventional radiocarbon age determinations, calibrated mean age ± 1σ and rates of accumulation, calculated using depth below surface, of the Wathayn shell mounds.

Mound WPSM number and sample		Lab Number	DeltaC^13^	CRA (BP)	Mean ± 1σ cal age (y BP)	Depth (cm)	Accumulation Rate (RA) for pairs of dates (cm/100 y)
50	S1	Wk-38204	–2.8	1541 ± 24	1207 ± 37	45	S1 to S2: 7.60 S2 to S3: 5.15
	S2	Wk-38205	–2.9	1268 ± 24	916 ± 39	30	
	S3	Wk-38206	–2.9	990 ± 25	653 ± 31	10	
53	S1	Wk-38211	–2.5	3612 ± 18	3634 ± 44	30	S1 to S2: 0.75
	S2	Wk-38212	–2.9	1326 ± 25	976 ± 39	10	
55	S2	Wk-35218	–2.4	3938 ± 36	4062 ± 67	75	S2 to S3: 13.33 S3 to S4: 6.04 S4 to S5: 0.99
	S3	Wk-35219	–2.2	3825 ± 25	3912 ± 51	55	
	S4	Wk-35220	–2.2	3501 ± 28	3498 ± 49	30	
	S5	Wk-35221	–2.9	1826 ± 27	1471 ± 47	10	
58	S1	Wk-35237	–3.4	1380 ± 21	1027 ± 44	60	S1 to S2: 7.14 S2 to S3: 6.23 S3 to S4: 6.47
	S2	Wk-35238	–2.9	1104 ± 28	747 ± 43	40	
	S3	Wk-35239	–3.2	683 ± 25	426 ± 40	20	
	S4	Wk-35240	–2.9	459 ± 25	194 ± 49	5	
63	S1	Wk-32285	–2.4	2660 ± 32	2492 ± 81	100	S1 to S2: 20.77 S2 to S3: 2.05
	S2	Wk-32286	–2.2	2402 ± 32	2179 ± 62	35	
	S3	Wk-32287	–2.1	1540 ± 31	1204 ± 42	15	
64	S1	Wk-32288	–2.3	2695 ± 33	2545 ± 78	35	S1 to S2: 38.46 S2 to S3: 11.11
	S2	Wk-32289	–2.5	2665 ± 32	2500 ± 81	30	
	S3	Wk-32290	–2.4	2649 ± 32	2474 ± 79	20	
65	S1	Wk-32291	–1.7	3193 ± 26	3142 ± 58	65	S1 to S2: 3.52 S2 to S3: 12.55
	S2	Wk-32292	–2.4	2718 ± 33	2574 ± 72	45	
	S3	Wk-32293	–2	2542 ± 32	2335 ± 53	15	
66	S3	Wk-28972	–2.5	2726 ± 39	2580 ± 76	90	S3 to S2: 40.82 S2 to S1: 38.46
	S2	Wk-28970	–2.1	2684 ± 29	2531 ± 77	70	
	S1	Wk-28969	–2	2590 ± 39	2401 ± 66	20	
67	S2	Wk-32295	–2.3	2516 ± 32	2295 ± 54	25	S2 to S1: 7.58
	S1	Wk-32294	–2.5	2456 ± 32	2229 ± 53	20	
68	S2	Wk-32297	–2	2539 ± 31	2330 ± 52	35	S2 to S1: 27.47
	S1	Wk-32296	–2.5	2468 ± 30	2239 ± 52	10	
69	S3	Wk-32300	–2.8	2696 ± 27	2549 ± 73	60	S3 to S2: 31.25 S2 to S1: 36.23
	S2	Wk-32299	–2.7	2657 ± 27	2485 ± 77	40	
	S1	Wk-32298	–2.3	2611 ± 28	2416 ± 60	15	
70	S4	Wk-32304	–2.3	3523 ± 34	3792±53	60	S4 to S3: 3.55 S3 to S2: 2.89 S2 to S1: 21.74
	S3	Wk-32303	–2.1	3024 ± 32	3228±60	40	
	S2	Wk-32302	–2.5	2784 ± 32	2882±44	30	
	S1	Wk-32301	–2.4	2749 ± 28	2836±36	20	
71	S3	Wk-32307	–2.2	2692 ± 27	2543±74	55	S3 to S2: -39.22 S2 to S1: 64.10
	S2	Wk-32306	–2.2	2733 ± 29	2594±66	35	
	S1	Wk-32305	–2.6	2700 ± 27	2555±72	10	
72	S3	Wk-32310	–2.6	2751 ± 27	2616±62	80	S3 to S2: -321.43 S2 to S1: 41.67
	S2	Wk-32309	–2.2	2762 ± 27	2630±61	35	
	S1	Wk-32308	–2	2742 ± 27	2,606±25	25	
73	S1	Wk-32311	–3.2	1852 ± 26	1503±50		
74	S1	Wk-32312	–2.1	2692 ± 33	2541±78	75	S1 to S2: -24.69 S2 to S3: -65.22 S3 to S4: 3.98
	S2	Wk-32313	–2.3	2758 ± 31	2622±64	55	
	S3	Wk-32314	–2.6	2793 ± 31	2668±55	25	
	S4	Wk-32315	–2.6	2513 ± 31	2291±53	10	
75	S1	Wk-28976	–2.2	1824 ± 37	1470±55	125	S1 to S2: -51.95 S2 to S3: 24.75 S3 to S4: 8.03
	S2	Wk-28975	–2.6	1884 ± 29	1547	85	
	S3	Wk-28974	–2.7	1800 ± 28	1446±46	60	
	S4	Wk-28973	–3.3	1359 ± 37	1010±51	25	
76	S1	Wk-28984	–2.2	2693 ± 39	2540±82	145	S1 to S2: 80.65 S2 to S3: 16.67
	S2	Wk-28979	–2.4	2607 ± 33	2416±64	45	
	S3	Wk-28978	–3.2	2485 ± 38	2254±58	18	
77	S1	Wk-28991	–2.1	2772 ± 37	2634±67	185	S1 to S2: 33.78 S2 to S3: 110.29 S3 to S4: 222.22
	S2	Wk-28990	–1.9	2708 ± 37	2560±78	160	
	S3	Wk-28988	–2.4	2659 ± 35	2492±83	85	
	S4	Wk-28986	–2.1	2642 ± 35	2465±80	25	
78	S3	Wk-32318	–2.1	2721 ± 30	2579±69	75	S3 to S2: 18.63 S2 to S1: 17.85
	S2	Wk-32317	–2.2	2610 ± 32	2418±64	45	
	S1	Wk-32316	–2.2	2503 ± 29	2278±51	20	
79	S2	Wk-32320	–2.6	2449 ± 30	2223±52	30	S2 to S1: 95.24
	S1	Wk-32319	–1.9	2423 ± 30	2202±55	10	
80	S3	Wk-32323	–2.6	2664 ± 32	2499±81	85	S3 to S2: 183.33 S2 to S1: 25.00
	S2	Wk-32322	–2.3	2647 ± 29	2469±77	30	
	S1	Wk-32321	–2.3	2605 ± 29	2409±58	15	
81	S1	Wk-35262	–2.4	2835 ± 22	2719±27	50	S1 to S2: 2.62
	S2	Wk-35263	–2.9	1738 ± 23	1381±44	15	
82	S1	Wk-35270	–3	1725 ± 23	1366±41	70	S1 to S2: -21.73 S2 to S3: 5.56 S3 to S4: 88.23 S4 to S5: 47.61 S5 to S6: -166.67 S6 to S7: -111.11 S7 to S8: 7.14
	S2	Wk-35271	–1.6	1745 ± 22	1389±45	65	
	S3	Wk-35272	–4.2	1646 ± 24	1299±30	60	
	S4	Wk-35273	–3.4	1627 ± 19	1282±27	45	
	S5	Wk-35274	–2.5	1603 ± 21	1261±30	35	
	S6	Wk-35275	–2.5	1610 ± 24	1267±31	25	
	S7	Wk-35276	–2.5	1620 ± 24	1276±31	15	
	S8	Wk-35277	–2.7	1473 ± 24	1136±46	5	
83	S1	Wk-35294	–3.2	2490 ± 25	2264±49	125	S1 to S2: -75.00 S2 to S3: 13.89 S3 to S4: 62.50 S4 to S5: 10.47 S5 to S6: 19.61 S6 to S7: 12.40 S7 to S8: 37.04 S8 to S9: 41.67 S9 to S10: 4.07 S10 to S11: 1.39
	S2	Wk-35295	–3.4	2506 ± 25	2284±48	110	
	S3	Wk-35296	–3	2477 ± 29	2248±51	105	
	S4	Wk-35297	–2.8	2459 ± 30	2232±51	95	
	S5	Wk-35298	–2.5	2305 ± 27	2041±50	75	
	S6	Wk-35299	–2.5	2262 ± 25	1990±50	65	
	S7	Wk-35300	–2.5	2159 ± 24	1869±43	50	
	S8	Wk-35301	–2.4	2136 ± 31	1842±50	40	
	S9	Wk-35302	–2.1	2105 ± 21	1806±44	25	
	S10	Wk-35303	–3.4	1894 ± 21	1560±44	15	
	S11	Wk-35304	–2.6	1195 ± 21	841±40	5	
90	S1	Wk-32324	–2.9	2164 ± 32	1875±50	130	S1 to S2: 40.00 S2 to S3: 7.87 S3 to S4: 31.25 S4 to S5: 17.14 S5 to S6: 14.18
	S2	Wk-32325	–3.3	2122 ± 30	1825±49	110	
	S3	Wk-32326	–2.3	2012 ± 31	1698±57	100	
	S4	Wk-32327	–2.8	1915 ± 32	1586±53	65	
	S5	Wk-32328	–1.9	1763 ± 29	1411±49	35	
	S6	Wk-32329	–2.7	1614 ± 31	1270±36	15	
91	S1	Wk-32331	–2.2	2540 ± 25	2331±44	40	S1 to S2: -142.86
	S2	Wk-32332	–1.7	2554 ± 32	2352±53	10	
92	S1	Wk-32333	–1.7	2485 ± 31	2256±53	30	S1 to S2: -53.57
	S2	Wk-32334	–1.8	2508 ± 30	2284±52	15	
94	S1	Wk-32335	–2.6	2752 ± 32	2614±66		
99	S1	Wk-38207	–2.7	704 ± 26	473±25		
103	S1	Wk-35338	–2.7	1015 ± 20	673±25		
104	S1	Wk-35339	–2.1	1147 ± 21	795±44	20	S1 to S2: 6.06
	S2	Wk-35340	–2.8	963 ± 21	630±30	10	
105	S3	Wk-35354	–2.1	2639 ± 20	2448±66	50	S1 to S2: -19.05 S2 to S3: 9.90
	S2	Wk-35347	–2.1	2685 ± 28	2532±76	34	
	S1	Wk-35348	–2.9	2455 ± 28	2229±51	4	
106	S3	Wk-35357	–2	4927 ± 25	5378±45	60	
	S2	Wk-35356	–2	661 ± 20	414±41	20	
	S1	Wk-35355	–1.6	2642 ± 24	2456±71	10	
109	S1	Wk-35360	–2.6	862 ± 19	561±31	10	
110	S1	Wk-35361	–2.3	698 ± 19	444±31	15	
111	S1	Wk-35362	–2.9	2125 ± 20	1829±42	25	S1 to S2: 8.77
	S2	Wk-35363	–2.3	1930 ± 24	1601±47	5	
122	S1	Wk-35365	–4	1189 ± 25	840	160	S2 to S3: 200 S3 to S4: 39.22 S4 to S5: 42.86 S5 to S6: 250.00 S6 to S7: 16.34 S7 to S8: 13.64 S8 to S9: -93.75 S9 to S10: -375.00 S10 to S11: 50.00
	S2	Wk-35366	–1.6	1620 ± 25	1276±31	150	
	S3	Wk-35367	–1.7	1615 ± 25	1271± 32	140	
	S4	Wk-35368	–2.6	1557 ± 25	1220±36	120	
	S5	Wk-35369	–3	1516 ± 22	1185±41	105	
	S6	Wk-35370	–2.9	1512 ± 27	1179± 44	90	
	S7	Wk-35371	–3.1	1379 ± 23	1026± 45	65	
	S8	Wk-35372	–3.5	1268 ± 25	916±39	50	
	S9	Wk-35373	–3.1	1281 ± 26	932±39	35	
	S10	Wk-35374	–3.2	1286 ± 17	936±31	20	
	S11	Wk-35375	–3.1	1258 ± 17	906± 34	5	
123	S1	Wk-35384	–3.1	1575 ± 23	1234±33	70	S1 to S2: -9.15 S2 to S3: 14.39 S3 to S4: 500.00
	S2	Wk-35385	–2.7	1753 ± 22	1398±45	55	
	S3	Wk-35386	–3	1601 ± 19	1259±29	35	
	S4	Wk-35387	–2.9	1597 ± 19	1256	20	
148	S3	Wk-38210	–3	1519 ± 29	1186±44	25	S3 to S2: -20.00 S2 to S1: 44.12
	S2	Wk-38209	–3.1	1546 ± 26	1211± 37	20	
	S1	Wk-38208	–2	1510 ± 27	1177± 45	5	
149	S1	Wk-38211	-1.7	2217 ± 34	1937±53		
150	S1	Wk-38226	–2.2	2564 ± 26	2362±46	50	S1 to S2: 30.49 S2 to S3: 45.45
	S2	Wk-38227	–2.3	2504 ± 28	2280±50	25	
	S3	Wk-38228	–2.4	2476 ± 28	2247± 51	10	
151	S2	Wk-38230	–2.7	1876 ± 18	1537± 43	30	S1 to S2: 7.66
	S1	Wk-38229	–2.5	1620 ± 22	1276± 29	10	
152	S3	Wk-38251	-3.8	1490 ± 24	1155±46	35	S3 to S2: 7.08 S2 to S1: -12.82
	S2	Wk-38250	-2.8	1526 ± 25	1194±40	30	
	S1	Wk-38249	-2.9	1195 ± 19	841±39	5	
153	S4	Wk-38271	–3.6	1164 ± 23	813±44		
	S3	Wk-38254	–3.2	1317 ± 26	968± 39		
	S2	Wk-38253	–2.7	1246 ± 27	989±43		
	S1	Wk-38252	–3.6	1225 ± 25	866±41		
154	S3	Wk-38257	–4	1048 ± 16	696±26	50	S3 to S2: 34.09 S2 to S1: -136.36
	S2	Wk-38256	–2.7	936 ± 18	608±31	20	
	S1	Wk-38255	–3.1	949 ± 17	619±30	5	
155	S3	Wk-38260	–2.7	1882 ± 31	1545±54	55	S3 to S2: 21.60 S2 to S1: 2.69
	S2	Wk-38259	–2.2	1740 ± 22	1383±44	20	
	S1	Wk-38258	–2.5	1365 ± 22	1011±41	10	
156	S1	Wk-38231	–2.3	2664 ± 24	2496±76		
157	S2	Wk-38233	–2.1	2301 ± 31	2387±51	20	S2 to S1: 4.27
	S1	Wk-38232	–1.9	2585 ± 28	2036±54	5	
158	S1	Wk-38234	–1.9	2432 ± 25	2211±51		

Mean ages are based on the Marine09 curve and a ΔR of –103±16 14C yr. S1 File contains the original data and calculations used to determine rates of accumulation. No accumulation rate results are provided for mounds with single ages. Determinations for WPSM106 are reported but without rates because of an age inversion. WPSM 153 depths were not recorded.

### Mound location and age

Mounds with age determinations around 2,500 cal BP are primarily located in the Wathayn Central part of the study area (Fig 3). Two mounds containing the oldest deposits in the study area (WPSM53 and WPSM55) are located in the Wathayn West area, while a third, WPSM70, is located in the Wathayn Central area. Mounds with deposits younger than 2,000 cal BP are located across the whole of the study area. There is no detectable pattern of shell deposit ages with geographic location, either in an upstream/downstream direction or with distance inland from the estuary.

### Mound accumulation rates

Table 2 shows accumulation rates calculated for the Wathayn shell mounds using the method of Stein et al. [54]. For all the reasons discussed above, these rates should be thought of as only approximate estimates of the true rates of mound accumulation. Therefore the accumulation rate calculations are analysed in relative terms only, by grouping the rates by speed of accumulation (i.e. slow (less than 10 cm/100 y), medium (greater than 10 and less than 100 cm/100 y), or rapid (greater than 100 cm/100 y)) and whether they are uniform or variable through the individual SMDs.

Some of the calculated accumulation rates in Table 2 return negative values. This occurs because, in the calculation of RA, DA is the difference in age between stratigraphically adjacent pairs of determinations. In some instances, the mean calibrated age of the stratigraphically higher determination is slightly older than the mean calibrated age of the stratigraphically lower determination. However, in these instances both means always fall within the one sigma confidence intervals. For example, for WPSM72, the RA result of -321.43 cm/100 y is negative because the stratigraphically lower date of 2,616±62 cal BP is slightly younger than the stratigraphically higher date of 2,630±61 cal BP. This result indicates that the rate of deposition is very rapid. For the analysis of relative deposition rates, only the magnitude of the accumulation rates are considered.

One group of SMDs exhibit relatively slow rates of accumulation. Six of these have only two age determinations (WPSM53, 67, 81, 104, 111, 157), therefore it is not possible to determine if accumulation rates varied within the individual mounds. However, for other mounds with more than two age determinations, changes in accumulation rates within each mound can be assessed. For example, WPSM50 and WPSM58 exhibit slow, relatively uniform accumulation rates (5.16–7.60 cm/100 y; Table 2), while the rates for WPSM55 (0.99–13.33 cm/100 y) and WPSM65 (3.52–12.55 cm/100 y) are more variable although still relatively slow. There does not appear to be any relationship between the overall age of mounds and rates of accumulation where the rates are relatively slow. For example, relatively slow rates are calculated for both young mounds like WPSM58 and for older mounds like WPSM55. Mounds that date to the period 2,000–3,000 BP also have slow rates of deposition.

Amongst the group of mounds with medium to rapid accumulation rates, WPSM79, WPSM91, and WPSM92 have high rates but these are based on only two age determinations per mound (Table 2). Of those with three or more age determinations, only WPSM150 exhibits a uniformly medium rate of accumulation, while twelve other mounds exhibit medium to rapid, but variable, rates of accumulation (WPSM72, 74, 75, 76, 77, 80, 82, 83, 90, 122, 123, 154). Mounds with rapid accumulation rates vary considerably in overall age (Table 2).

Other mounds in the Wathayn area exhibit medium rates of accumulation but also demonstrate a mix of both uniform (e.g., WPSM66, 69, 71, 78) and variable (e.g., WPSM63, 64, 70, 105, 148, 152, 155) accumulation rates. Two other mounds, WPSM68 and WPSM151, exhibit medium to low deposition rates but these are based on only two age determinations per mound. Mounds with medium rates of deposition tend to be older than 2,000 BP, with the exceptions being WPSM148, WPSM151, WPSM152, and WPSM155.

### Mound age and structure

Age determinations from the six shell mounds with multiple sets of samples collected from the centre to the outside edge of the feature (Table 3) indicate that, in some cases, there exists an older core deposit of shells completely buried both vertically and laterally by more recent deposits of shells. This internal pattern of shell ages is consistent with a model of mound formation where an initial deposit of shells is progressively buried by subsequent deposits, with shells from those subsequent deposition events migrating down the outside of the mound as the shells come to rest at their typical angle of repose. Under such a model, shells from the top and at the periphery of the mound should be of similar age, since each deposit would be draped over the previous one (see also Fig 3 in [59] for an example). For WPSM90, rates of deposition calculated at the centre of the mound are fastest and these reduce towards the edge of the mound (Table 3). For WPSM70, however, the older core of the mound aggraded more slowly than the younger mantle. This is also the situation for WPSM105. WPSM83 shows a more complex pattern, with rapid accumulation at the core of the mound but more variable rates of accumulation at higher levels in the mound. Toward the centre of this mound, these higher levels exhibit relatively slow rates of accumulation while accumulation rates are more rapid toward the periphery of the mound. WPSM151 also exhibits highly variable accumulation rates, with both relatively slow and relatively rapid rates in the centre and periphery of the mound as well as at the base and toward the top of the mound. For WPSM63, rates are relatively rapid at the base of the mound toward the centre but slower and more variable towards the periphery of the mound and near the mound surface. Further details of the relationships among mound size, shape, internal structure and age for the Wathayn mounds, based on the results of LiDAR survey, are reported in Larsen *et al*. [29].

**Table 3:** Conventional radiocarbon age determinations, calibrated mean age ± 1σ and rates of accumulation for shell mounds at Wathayn where multiple column samples were obtained along the length of the excavated trench.

Provenance (Mound WPSM number, distance of sample from mound centre and sample number)			Lab number	DeltaC^13^	CRA (BP)	Mean ± 1σ cal age (y BP)	Depth (cm)	Accumulation Rate (RA) for pairs of dates (cm / 100 y)
63	1.5 m	S4	Wk-33465	–2	2681 ± 31	2526±79	94	S4 to S5: 17.05 S5 to S6: 23.68 S6 to S7: 1.36
		S5	Wk-33466	–2	2496 ± 31	2268±53	50	
		S6	Wk-33467	–2.5	2384 ± 30	2154±64	23	
		S7	Wk-33468	–3.5	1532 ± 31	1197±43	10	
	3 m	S8	Wk-33469	–2.2	2644 ± 31	2465±77	77	S8 to S9: 19.13 S9 to S10: 18.18 S10 to S11: 3.29
		S9	Wk-33470	–1.9	2553 ± 31	2350±51	55	
		S10	Wk-33471	–1.9	2469 ± 30	2240±52	35	
		S11	Wk-33472	–2.1	1833 ± 28	1479±49	10	
	4.7 m	S12	Wk-33473	–2.3	2365 ± 28	2123±62	47	S12 to S13: 3.05 S13 to S14: 3.64
		S13	Wk-33474	–2.7	1958 ± 30	1631±52	32	
		S14	Wk-33475	–2.8	1473 ± 25	1136±47	14	
	6 m	S15	Wk-33476	–2.4	2514 ± 31	2292±53	27	S15 to S16: 6.37
		S16	Wk-33477	–1.7	2291 ± 30	2025±53	10	
	7.5 m	S17	Wk-33478	–1.8	2542 ± 32	2335±53		
70	1 m	S5	Wk-33479	–2	3571 ± 29	3582±53	52	S5 to S6: 1.94 S5 to S6: 5.78 S5 to S6: 13.08
		S6	Wk-33480	–2.4	3067 ± 32	2965±63	40	
		S7	Wk-33481	–2.1	2858 ± 32	2740±36	27	
		S8	Wk-33482	–2.1	2765 ± 29	2633±62	13	
	3 m	S9	Wk-33483	–2.5	3051 ± 32	2943±61	37	S9 to S10: 4.90
		S10	Wk-33484	–2.3	2734 ± 27	2596±65	20	
	4.5 m	S11	Wk-33485	–2.5	2837 ± 32	2720±39	30	S11 to S12: 13.51
		S12	Wk-33486	–2.2	2748 ± 33	2609±67	15	
83	2 m	S20	Wk-35313	–3.1	2433 ± 21	2212±49	109	S20 to S21: -80.00 S21 to S22: 44.74 S22 to S23: 7.81 S23 to S24: -57.14 S24 to S25: 27.59 S25 to S26: 9.52
		S21	Wk-35314	–2.9	2453 ± 28	2227±51	97	
		S22	Wk-35315	–2.1	2409 ± 23	2189±54	80	
		S23	Wk-35316	–2.6	2159 ± 26	1869±45	55	
		S24	Wk-35317	–2.3	2177 ± 26	1890±44	43	
		S25	Wk-35318	–2.8	2128 ± 29	1832±48	27	
		S26	Wk-35319	–3.6	1986 ± 29	1664±54	11	
	4 m	S32	Wk-35325	–2.2	2178 ± 29	1891±47	51	S32 to S33: 19.05 S33 to S34: 7.22
		S33	Wk-35326	–2.9	2049 ± 29	1744±50	23	
		S34	Wk-35327	–2.3	1807 ± 16	1453±40	2	
90	1.5 m	S7	Wk-33487	–3.1	2101 ± 32	1805±51	110	S7 to S8: 22.22 S8 to S9: 24.32 S9 to S10: 11.76
		S8	Wk-33488	–2.4	2021 ± 31	1710±56	90	
		S9	Wk-33489	–2	1868 ± 25	1525±50	45	
		S10	Wk-33490	–2.4	1614 ± 31	1270±36	15	
	3 m	S11	Wk-33491	–2.2	2198 ± 32	1914±50	110	S11 to S12: 10.31 S12 to S13: 13.16
		S12	Wk-33492	–3.1	2029 ± 33	1720±57	90	
		S13	Wk-33493	–3.1	1733 ± 31	1378±48	45	
	4.5 m	S14	Wk-33494	–2.3	2144 ± 32	185151	70	S14 to S15: 10.09
		S15	Wk-33495	–2.6	1758 ± 31	1405±50	25	
	6 m	S16	Wk-33496	–2.5	1788 ± 32	1435±49	40	S16 to S17: 7.55
		S17	Wk-33497	–2.4	1445 ± 32	1114±52	15	
105	1.5 m	S3	Wk-35349	–3	2659 ± 20	2485±73	29	S3 to S4: 2.07
		S4	Wk-35350	–2.7	1733 ± 26	1376±45	6	
	3 m	S5	Wk-35351	–2.4	2637 ± 28	2451±72	28	S5 to S6: 1.55
		S6	Wk-35352	–2.9	1494 ± 19	1161±43	8	
	4.5 m	S7	Wk-35353	–2	2507 ± 20	2288±43		
151	50 cm	S3	Wk-35235	–3.5	2352 ± 20	2099±51	249	
	150 cm	S6	Wk-35238	–2.6	2272 ± 28	2002±52	320.325	S6 to S5: 3.79 S5 to S4: 9.51
		S5	Wk-35237	–2.5	1859 ± 18	1513±45	301.79	
		S4	Wk-35236	–2.5	1425 ± 17	1083±42	260.89	
	300 cm	S9	Wk-35241	–2.6	2171 ± 26	1883±44	300	S9 to S8: 6.03 S8 to S7: 14.55
		S8	Wk-35240	–2.3	1900 ± 25	1568±47	281	
		S7	Wk-35239	–2.1	1709 ± 18	1348±34	249	
	450 cm	S11	Wk-35243	–2.3	1830 ± 20	1475±43	287.59	S11 to S10: 2.18
		S10	Wk-35242	–2.5	929 ± 24	602±32	268.55	
	600 cm	S12	Wk-35244	–2.8	1599 ± 21	1257±30	263	

Mean ages are based on the Marine09 curve and a ΔR of –103±16 14C yr. S1 File contains the original data and calculations used to determine rates of accumulation.

## Discussion

The central processing of large numbers of *T*. *granosa* in northern Australia by indigenous people led, at times, to the generation of large volumes of shell valves that required disposal. The process of disposal most evident in the archaeological record was the formation of shell mounds. Rapid shell accumulation rates sometimes resulted in the formation of very large shell mounds. However, variability in accumulation rates resulted in mounds of different sizes and shapes. Once discarded, the shell as well as the shell deposit was subject to changes depending on the local micro-environment. Because both shell accumulation and post-depositional deformation and diagenesis are time dependent, time is a factor in both the numerator and denominator in commonly used accumulation rate calculations, complicating how the rate of accumulation in shell mounds should be interpreted.

The results obtained from the Wathayn study area illustrate how rates of accumulation vary in ways predicted by our earlier hypotheses about how mounds accumulate. The mounds we dated vary considerably in age, from 4,000 cal BP to within the last 500 years. Many of the shell mounds have ages around 2,000–2,500 cal BP. Rates of deposition vary considerably, from less than 10 to several hundred centimetres per 100 years. Mounds with ages that fall in the range 2,000–3,000 cal BP accumulated somewhat more rapidly than mounds before and after this period but there is little spatial consistency in mound accumulation rates from any time period and, in some cases, mounds located adjacent to each other accumulated at different rates.

For many of the mounds we studied, the available radiocarbon determinations indicate variable rates of accumulation. Mounds with uniform but slow rates of accumulation are rare. For a larger number of mounds, rates of accumulation change markedly within a single mound. For some, rates of accumulation are high at the base of the mound and low at the mound surface, and for others this difference is reversed.

There is also a variable relationship between rates of accumulation and mound structure. In some mounds, an older core is covered by more recent deposits, with covering layers of shell at both the centre and periphery showing similar ages. For other mounds, the core of the mound accumulated more rapidly than deposits on the periphery. The situation is reversed in one of the mounds, where deposits at the centre accumulated more slowly than those on the periphery. In other cases, the pattern of accumulation is more complex, with mounds showing both relatively slow and rapid rates of accumulation at the centre and on the periphery of the mound as well as at the base and toward the top of the mound.

Variability in the rate of shell mound accumulation is not limited to mounds in our Wathayn study area. Dates obtained from soil and bone collagen samples from shell mounds by Pluckham and colleagues [60] indicate periods of slower and of more rapid accumulation, with rates calculated following Stein et al. [54]. Thompson et al. [61] use a similar approach but also caution that midden accumulation cannot be assumed to be a linear process. The ultimate intent of these studies (as well as ours) is to draw behavioural inferences from the way mounds accumulated–how many people were involved and over what duration and, in the case of the Thompson study, how mounds may have been reworked. The difficulty we all face–and what is clear from our study–is that mounds need to be considered on a case by case basis to more fully understand the dynamics of shell accumulation. As we have shown for one of the mounds at Wathayn [30], the chemistry of mounds, and therefore mound micro-environments, change with depth, therefore shell diagenesis needs to be assessed to understand how this has influenced deposit volume. Detecting variable rates of deposition is also dependent on the number of dates obtained and how samples were selected. At Wathayn, we selected samples based on the stratigraphic architecture of the mounds. However, for each of the mounds, samples were obtained from only one narrow trench excavated to the mound centre. Depending on how shells were deposited across an entire mound, there is the potential for a different trench excavated into the same mound to produce different rates of accumulation. Some indication of the potential for such variability is provided by the range of results we obtained when samples were obtained from along the length of the excavated trench. Even using a 1 m wide trench, results indicate that a simple “layer cake” model of shell accumulation cannot be assumed for all mounds. If we are correct in our proposition that the primary factor responsible for mound formation is the need to bring large quantities of molluscs with the size and shell-to-meat–weight ratios of *T*. *granosa* to a central place to process them with fire, then it is possible to imagine shells accumulating at different rates in different parts of a mound, something that would be very difficult to detect given commonly used sampling techniques based on the excavation of squares or trenches, or extraction of cores.

As discussed above, accounts of shell mound formation around the world provide a variety of behavioural explanations for mound formation based on mound size and shape, and rate of accumulation. However, the results of our Wathayn study suggest that caution is needed when comparing shell mound form and age.”Dating” mounds is not as simple as it may appear, given the ways in which deposits both accumulate and are transformed through time. The lesson from the Wathayn study is that the present-day form of shell mounds may hide a complex formational history. If the situation at Wathayn is in any way typical of other shell mounds, then a great deal of care is needed in assessing regional chronologies and in the interpretation of mound functions. Both the substantive dating results from Wathayn, and the evidence of variability in rates of accumulation within and between mounds, indicate that simply taking lots of “spot” dates from a group of mounds within the region at large, as advocated, for example, by Morrison [25], may lead to unreliable conclusions about the chronology of the shell mounds. The Wathayn results also indicate the presence of mounds with deposits dating to the period 1,800–2,350 cal BP, which appears to refute Morrison’s suggestion that there was a low probability of mound formation in the Albatross Bay region during that period. The issue here is whether the Wathayn results represent a true difference in the chronology of mound formation compared to other groups of mounds in the wider Albatross Bay region, or whether the chronological “gap” in mound accumulation apparent elsewhere is simply the result of using an unrepresentative sample of dates for other groups of mounds. A large number of mounds were excavated and dated from one geographic location at Wathayn, whereas Morrison and other researchers working in the Albatross Bay region used dates obtained from single mound excavations and, in some cases, small shell scatters widely spaced across the region. More than this, however, we need to assess shell mound formation processes from Wathayn and from other locations in Albatross Bay in relation to their local depositional environments. Mound geochemistry and studies of shell fragmentation, like those that we are currently undertaking [28, 29, 30, 62], are important in understanding what might be termed “whole mound chronologies”; that is, the apparent variability in rates of accumulation from different parts of a mound. Only then can we begin to interpret what the shell mounds might represent in terms of population size, economic behaviour or other potential cultural and symbolic significance to their creators.

## Conclusion

All of the 158 shell mounds from Wathayn, located along a 5 km stretch of the Embley River near Weipa in far north Queensland, Australia, were investigated in this study, although not all were dated. As a result, we have pushed back the chronology of shell mound formation in the Weipa area 1,500 years earlier than identified in previous studies. Mound accumulation in the period 1,800–2,300 cal BP is indicated, a period previously identified as a time when there was a low probability of mound formation. In addition, rather than simply seeking to refine phases of occupation in the region, we utilised the large number of dated samples from the mounds to investigate variability in the rates of accumulation both within and between mounds. Results indicate that there is considerable variability in the rate of mound formation even among mounds from one limited geographic area. Results of this study also raise questions about how rates of shell mound accumulation should be calculated, since both accumulation and diagenesis are time-dependent processes that feature on both sides of the accumulation rate equation. Our results also raise the issue of how to sample shell mounds to understand potential variability in shell accumulation rates, and indicate that the excavation and sampling strategy we used needs to be extended to fully address this issue. This requirement poses formidable logistical challenges, given the number and volume of shell mounds in many regions and the huge quantities of shell deposits that might have to be sampled. Excavating small samples by bucket and auger, as advocated by Cannon [63, 64] for the shell mounds of British Columbia, offers one method of efficiently obtaining large numbers of samples from many mounds, but is likely to run into the problem identified here of highly variable rates and patterns of individual mound formation. If the Wathayn results are typical of other regions with numbers of shell mounds, more attention will need to be given to the particular circumstances of individual mound formation when seeking to draw behavioural inferences from the rates at which mounds accumulate.

## Supporting information

S1 File Rates of accumulation calculations for Wathayn shell mounds. (XLSX)
